# Lumbosacral spinal cord epidural stimulation improves voiding function after human spinal cord injury

**DOI:** 10.1038/s41598-018-26602-2

**Published:** 2018-06-06

**Authors:** A. N. Herrity, C. S. Williams, C. A. Angeli, S. J. Harkema, C. H. Hubscher

**Affiliations:** 1 Kentucky Spinal Cord Injury Research Center, University of Louisville, Louisville, KY USA; 2 Department of Neurological Surgery, University of Louisville, Louisville, KY USA; 3 Department of Urology, University of Louisville, Louisville, KY USA; 4 Frazier Rehab Institute, Louisville, KY USA; 5 Department of Anatomical Sciences and Neurobiology, University of Louisville, Louisville, KY USA

## Abstract

Deficits in urologic function after spinal cord injury (SCI) manifest both as a failure to store and empty, greatly impacting daily life. While current management strategies are necessary for urological maintenance, they oftentimes are associated with life-long side effects. Our objective was to investigate the efficacy of spinal cord epidural stimulation (scES) as a promising therapy to improve bladder control after SCI. A bladder mapping study was undertaken for sixteen sessions over the course of four months in an individual with chronic, motor complete SCI. Varying combinations of stimulating cathode electrodes were initially tested during filling cystometry resulting in the identification of an effective configuration for reflexive bladder emptying at the caudal end of the electrode array. Subsequent systematic testing of different frequencies at a fixed stimulus intensity and pulse width yielded lowest post-void residual volumes at 30 Hz. These stimulation parameters were then tested in four additional research participants and found to also improve reflexive voiding efficiency. Taken together with SCI studies on step, stand, voluntary motor control and cardiovascular regulation, these findings further corroborate that scES has an all-encompassing potential to increase the central state of excitability, allowing for the control of multiple body functions, including the urological system.

## Introduction

Following spinal cord injury (SCI), volitional control to all or a portion of the lower urinary tract is impaired. Initially, there is a period of bladder areflexia and urinary retention followed by the emergence of spinally-mediated voiding reflexes and bladder hyperreflexia^1^. Subsequently, involuntary, uninhibited reflex detrusor contractions occur at low volumes of stored urine and can lead to loss of continence. Additionally, as the detrusor muscle contracts, the external urethral sphincter simultaneously reflexively contracts, causing detrusor-sphincter dyssynergia^2^. Uncoordinated activity between the bladder muscle and its sphincter produces high intravesical pressure that can lead to vesicoureteral reflux, potentially damaging both the lower and upper urinary tracts^3,4^. Therefore, reflex or spontaneous voiding that includes suprapubic tapping and an external condom collection system or urinary pads is usually not recommended. However, a recent finding of improved voiding efficiency and low-pressure voiding was obtained following injection of Botulinum toxin into the sphincter^5^. This method is gaining acceptance as an effective way to conduct reflex or spontaneous bladder voiding following SCI. Thus the most common, long-term management of neurogenic disorders of micturition commonly includes clean intermittent catheterization and conservative pharmacological therapy to decrease bladder over-activity, high intravesical pressure, and/or proximal urethral resistance while increasing bladder capacity^6,7^. Despite the necessary conventional approaches to manage lower urinary tract dysfunction, commonly prescribed anti-cholinergics have side effects such as dry mouth and constipation^7^ that exacerbate existing bladder/bowel issues. Chronic daily catheterization is also associated with scarring and strictures, cystitis, formation of false tracts, frequent urinary tract infections, and renal disease^8^. Additionally, in individuals with cervical injuries having compromised hand function, self-intermittent catheterization is not always a viable option^8^ leading to caregiver dependence or indwelling catheter management. Thus, there is a need for additional measures with fewer side effects, but ones that also target functional bladder recovery, as bladder dysfunction still ranks among the top disorders affecting quality of life^9–11^.

Locomotor training (LT) is one intervention which has emerged as a safe and effective therapy for post-SCI motor deficits and has been shown to provide additional benefits to autonomic function^12–15^. Furthermore, the combination of LT (step and stand training) plus spinal cord epidural stimulation (scES) has not only enhanced the execution of motor tasks in clinically motor complete SCI individuals but has also resulted in improved physiologic outcomes such as temperature regulation, bladder, and sexual function^16,17^. Despite preliminary reports on enhanced urologic outcomes following scES, the effect of scES alone on bladder function in humans has not been specifically targeted, as the stimulation parameters used in our center thus far have been directed toward recovery of motor and cardiovascular function^18–20^. In the current study we initially tested the effects of scES specifically for urinary bladder function (UB-scES) in a clinically motor complete SCI individual. Mapping using different anode/cathode configurations, spinal cord locations, and stimulation frequencies for bladder effects during repeated cystometry evaluation revealed an effective lumbosacral scES electrode configuration that improved reflexive voiding efficiency in this individual. This configuration was then re-tested in four additional individuals (3 AIS A, 1 AIS B), already implanted with spinal cord epidural stimulators. Our goal was to demonstrate efficacy of scES to excite the spinal cord circuitry at the lower lumbosacral region and facilitate neural output to the bladder to improve storage and/or elimination.

## Materials and Methods

### Research Participant

The participant, B23, is a 31-year old male (age at first pre-training Urodynamics assessment), who was enrolled in a research study conducted at the University of Louisville investigating the effects of activity-based training in combination with scES on the recovery of lower limb motor function. As part of that study, he received an epidural spinal cord stimulator (Medtronic, RestoreAdvanced) and a 16-electrode array (Medtronic, 5-6-5 Specify) that was surgically placed at the spinal segments L1-S1, 3.3 years after traumatic SCI because of a mountain biking accident (see Table 1 for project timeline). Prior to and following implantation of the spinal cord epidural stimulator, he received intensive LT (stand/step and stand/step in combination with scES, respectively). Results assessing the impact of LT on B23 have previously been published^15^. The current bladder mapping experiment was conducted after B23 completed the LT + scES study on the recovery of lower limb motor function and does not represent selective data pooling from a larger study. Table 1 provides a context for how the bladder mapping project was initiated. The participant provided written, informed consent, and the research was approved by the Institutional Review Board (University of Louisville, Louisville, KY). All research activities were performed in accordance with the guidelines and regulations of the Institutional Review Board.

**Table 1:** Timeline of therapy progression for participant B23.

**Time point**	Pre Implant Post Training LT	Surgery	Post Implant Pre Training LT	Post Implant Mid Training LT	Post Implant Post Training LT	Home Trainig	6 month Follow Up
Training Sessions	40 stand 40 step			40 Stand-scES 40 Step-scES	40 Stand-scES 40 Step-scES	Stand-scES	Stand-scES
Frequency	Each on same day; 5 d/wk			Each on alternating days; 5 d/wk	Each on same day; 5 d/wk	Daily; 5 d/wk	Daily; 5 d/wk
Assessment	Urodynamics		Urodynamics	Urodynamics	Urodynamics	**Bladder Mapping**	Urodynamics
Duration	3 months	4 weeks		5 months	4 months	1 month	6 months

d/wk, days per week; LT, Locomotor Training; scES, spinal cord epidural stimulation.

### Clinical Evaluation

Research participant B23 received a clinical evaluation prior to this study to assess motor and sensory status. Two clinicians independently performed the International Standards for Neurological Classification of Spinal Cord Injury^21,22^ in order to classify the participant’s injury using the ASIA (American Spinal Injury Association) Impairment Scale (AIS). Following the assessment, he was classified as AIS B (pinprick and light-touch present below the lesion), with a neurological level of injury at C5. A physical examination also was performed by a clinician for medical clearance, ensuring participation safety using the following inclusion criteria: (1) stable medical condition; (2) epidural stimulator implanted at the lumbosacral spinal cord; and (3) bladder dysfunction as a result of SCI. Note that B23 had never received Botox injections for management of bladder dysfunction but had a suprapubic catheter. Individuals with suprapubic catheters tend to have low capacities, which was the case for B23. Multiple fill-void cycles could thereby be accomplished in each 90-minute cystometry session (a common fill rate of 20 ml/minute was used).

### Activity-based training

Prior to epidural stimulator implant, research participant B23 underwent 80 sessions of LT (Table 1), which included stand and step training, with the goal of achieving the positive adaptations induced by activity-based training alone before the beginning of LT with scES. Following implantation, participant B23 continued 160 sessions of locomotor training (stand plus step training). Stand and step training were performed on separate days for the initial 80 sessions, alternating the intervention between days. Following the midpoint, a second session per day was added. One day a week, every 2–3 weeks, was added until reaching 5 days per week of stand and step training on the same day, alternating the training intervention between the morning and afternoon, with each session lasting 1 hour, 5 days per week and always performed with scES.

### Urodynamics

Data were obtained from standard urodynamic evaluations with recommendations from the International Continence Society^23–26^. All studies were performed by the same registered nurse using the Aquarius® LT (Laborie, Williston, VT) urodynamic investigation system. Bladder medication (10 mg Oxybutinin twice a day) was ceased 24 hours prior to every urodynamics session. The procedure was discussed with the research participant, including any risks and potential side effects not limited to infection and/or bleeding. Cystometry was performed in the supine position via a single sensor, dual channel catheter (7 Fr, T-DOC® Air-Charged™, Laborie, Williston, VT) with continuous filling of sterile, body-temperature water (37 °C) at a fixed slow rate of 20 ml/min. Abdominal pressure was measured via a rectal balloon catheter (7 Fr, T-DOC® Air-Charged™, Laborie, Williston, VT). Pelvic floor electromyography (EMG) (Neotrode II, Laborie, Williston, VT) was recorded using surface patch EMG electrodes and a grounding pad was placed on a bony prominence, usually the hip or knee. Detrusor pressures were calculated by subtracting the intra-abdominal pressure from the intra-vesical pressure. B23 was asked to cough to verify intra-abdominal catheter position, instructed to communicate filling sensations as follows: First sensation of fullness (FSF) – the first sense that there is fluid in the bladder; First desire (FD) – the feeling that one would void at the next convenient moment; Strong desire (SD) – a compelling need to void that is less comfortable to postpone; Capacity (C) – the feeling that voiding cannot be delayed any longer. The volume of water and bladder pressure was recorded. Uninhibited bladder contractions also were identified. The research participant was asked to empty his bladder while voiding bladder pressures were recorded.

Blood pressure (BP), heart rate (HR) and oxygen saturation were recorded every minute during urodynamics using an automated sphygmomanometer (DinamapV100; GE Medical Systems, Fairfield, CT). Baseline BP recordings were obtained in the supine position prior to urodynamic testing. Any signs and self-reported symptoms of autonomic dysreflexia were documented and observed throughout testing. Bladder filling was ceased if any of the following conditions were observed: (1) spontaneous urine leakage, (2) infused volume ≥600 mL, (3) sustained high intravesical filling pressure ≥40 cmH_2_O (if present for greater than 15 seconds) or (4) autonomic dysreflexia as evidenced by a sudden rapid rise in blood pressure from baseline and/or intolerable symptoms (such as a pounding headache). A post-fill BP recording was captured to ensure BP values returned to baseline.

### Spinal Cord Epidural Stimulation

Spinal cord epidural stimulation targeting the urinary bladder (UB-scES) was administered during cystometry through a multi-electrode array (Medtronic Specify 5-6-5, Restore ADVANCED) implanted in the epidural space over spinal cord segments L1–S1 (at vertebral levels T11–T12)^16^. An implanted package containing stimulating circuits, rechargeable battery, and wireless communication activates the electrodes (16 platinum electrodes arranged in three columns of 5-6-5). The pattern of electrically active electrodes, as well as electrode voltage, stimulating frequency, and stimulating pulse width can be remotely programmed. Guidelines for selecting electrodes were based on our previous work^16,20^. Briefly, the participant underwent 16 urodynamic sessions, in which a maximum of 6 fill/void cycles were performed on each occasion. The establishment of stimulating parameters was initiated by using a global configuration, which is defined by (1) selecting cathodes (−) and anodes (+) at opposite ends of the array in order to generate either a caudal or rostral flow of current. (2) Stimulating cathode electrodes at the caudal end of the array targeted the lower lumbosacral region of the cord for bladder emptying. (3) Electrode configurations were then modified by reducing the distance between the cathodes and anodes. (4) Using a fixed frequency (beginning at 5 Hz) and pulse width (450 µs), voltage was ramped up slowly (0.1 V increments) while the effects on motor evoked responses were monitored. The ramp up on voltage continued until muscle contraction (present in one or multiple muscles: gluteus maximus, vastus lateralis, biceps femoris, tibialis anterior, and soleus) was present as a result of the stimulation (then lowered 0.1 V - stimulation intensity targeted to be just below motor response threshold). The bladder was then filled with sterile water at a fixed 20 ml per minute rate while the stimulation was on and bladder pressure was monitored. Using the identified electrode configuration, three separate urodynamic sessions were subsequently performed to evaluate the effects of varying frequency (in the order of: no scES, 5, 15, 30, 45, or 60 Hz) on voiding efficiency values. Each session always included one cycle without the use of scES for baseline comparison. To distinguish between a targeted stimulation effect on bladder emptying versus repeated cystometry subsequently resulting in larger bladder volumes, a fill cycle without the use of UB-scES was also conducted at the end of a frequency response testing session. The time for each fill/void cycle (approximately 6 minutes) as well as the time interval between each fill/void cycle (approximately 5 minutes) was kept consistent throughout testing. Given the intensity ramp-up phase of UB-scES added time prior to filling, the interval time period from testing with scES to no scES (i.e. when frequency was reversed and no scES was tested last) was also set at 5 minutes to be consistent. Testing of B23’s effective voiding efficiency electrode configuration was conducted in four additional scES implanted research participants (A60 - T4 AIS A; A68 - C5 AIS A; B21 – C4 AIS B; A41 – C4 AIS A) for three fill-void cycles that included one without stimulation and two using different frequencies (selected based upon the Results from B23). In this instance, the fill cycle order was: no scES, UB-scES at 5 Hz, UB-scES at 30 Hz.

### Analysis

Bladder capacity was calculated as the volume of leaked or voided fluid plus any residual amount removed from the bladder. Voiding efficiency was calculated as: [volume voided/(volume voided + residual volume) × 100]. Compliance was calculated by dividing the volume change (ΔV) by the change in detrusor pressure (ΔPdet) during that change in bladder volume and was expressed in ml/cm H_2_O^27^. The intravesical pressure (Pves) at which involuntary expulsion of water/urine from the urethral meatus was observed was considered the detrusor leak point pressure (DLPP). Maximum detrusor pressure (MDP) was identified as the peak detrusor pressure during the voiding phase of the cystometrogram. Detrusor pressures were calculated by subtracting the intra-abdominal pressure from the intra-vesical pressure.

### Data Availability

All relevant data generated and analyzed are included in this published article.

## Results

**Figure 1:**
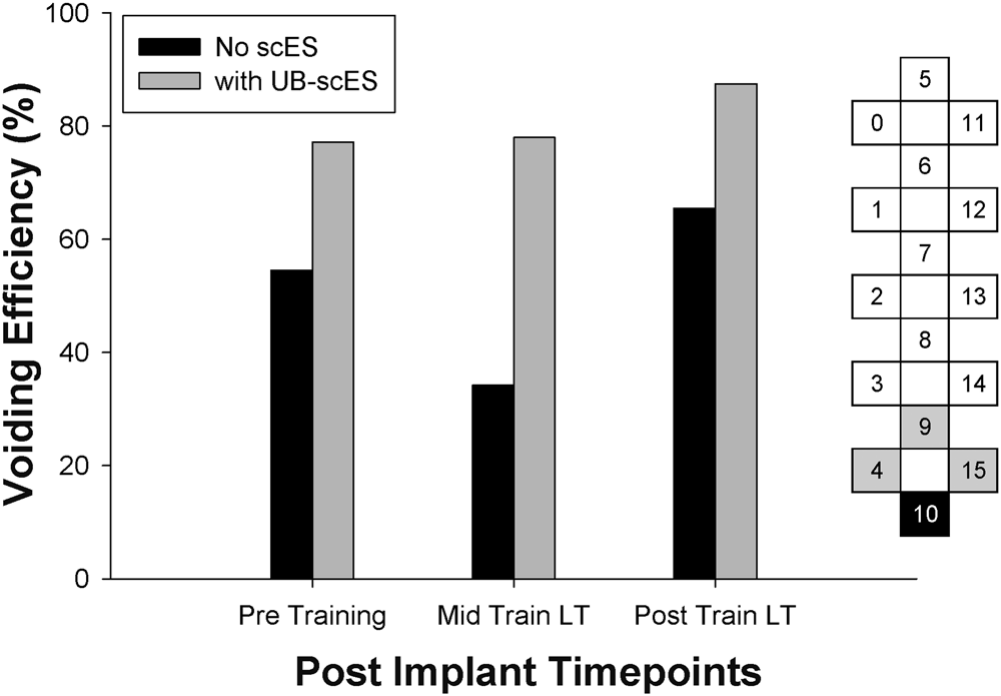
Improvements in voiding efficiency using UB-scES. Voiding efficiency values are presented, with and without the use of UB-scES during filling cystometry. Three separate urodynamic assessments were performed following epidural stimulator implantation: pre-training, mid-training, and at the post-training time point. Each time point tested bladder emptying with and without scES. The electrode configuration used during bladder filling cystometry is illustrated to the right of the graph (16-electrodes numbered from 0–15 in the 5-6-5 array). A narrow configuration was initially selected with cathode stimulation (black (−) electrode, 30 Hz, 450 µs) isolating the distal array and anode selection (grey (+) electrodes, inactive electrodes in white) surrounding the targeted stimulation region. At each time point, voiding efficiency improved with scES. These results are for one participant.

Cystometry was conducted just prior to training and repeated just after completion of the 80 LT training sessions. Following 80 LT sessions, B23’s voiding efficiency improved from 21.9% to 68.5% by the post-training time point (data published in^15^). Cystometry was then repeated after epidural stimulator implant, just prior to the next training period (post implant, pre-training), again at mid-training and finally at the post-training time point. The first fill/void cycle was performed without scES and then immediately followed by a fill/void cycle using UB-scES. Selection of electrode configurations targeted the lower region of the array to generate a stimulation zone near the pelvic parasympathetic outflow. Figure 1 illustrates the increased gains in voiding efficiency with the use of UB-scES targeting urinary bladder emptying as compared to voiding efficiency values achieved without the use of scES at each post-implant time point.

At B23’s post-implant, pre-training time point, the initial fill/void cycle was performed without the use of scES, indicating maintenance of the acquired pre-implant voiding efficiency value near 60%. The added effect of stimulation alone is immediately apparent as the use of UB-scES in the next fill/void cycle increased voiding efficiency to 77%. By post-implant, post-training, voiding efficiency reached 87.5%, approximating the recommended bladder emptying range established by the International Continence Society (ICS) guidelines^28^ (>90% or less than 25 ml post-void residual volume).

**Figure 2:**
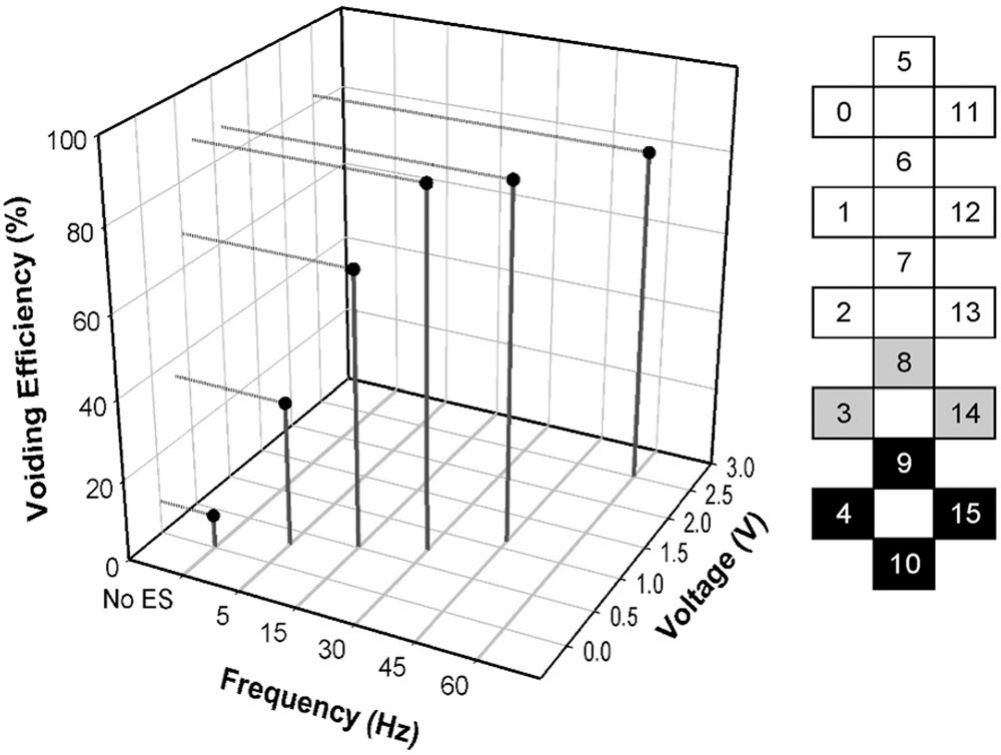
Bladder mapping isolates an effective voiding efficiency configuration and frequency. Bladder voiding efficiency responses to varying UB-scES frequencies is represented by the average of 6 fill/void reflex cycles from 3 separate urodynamic sessions (total of 18 cycles). One cycle from each session was performed without the use of scES reflecting low efficiency (8.3 ± 4.6%). The electrode configuration used during bladder filling cystometry is illustrated to the right of the graph with an expanded cathode selection (black (−) electrodes) targeting the lumbosacral region (anode (+) selection in grey, inactive electrodes in white). Frequency, varied at voltage sub-threshold levels, was most effective for voiding efficiency at 30 Hz (88.1 ± 1.1%). Note that voiding efficiency ≥90% is considered within normal limits for lower and upper urinary tract preservation^28^. These results are for one participant.

Given the extent of the post-implant increases in voiding efficiency values with the use of UB-scES in B23, we designed bladder mapping assessments aimed at enhancing lower lumbosacral stimulation for bladder emptying. Various simulating electrode combinations (including at the upper end of the array) were tested over a period of 4 months involving 16 sessions as only one configuration could be examined per fill-void cycle. Note that the participant was not engaged in any LT over the 4-month period. Testing included an expansion of the number of stimulating electrodes at the lower end of array (Fig. 2 diagram) from the combination used initially (Fig. 1) which yielded the greatest voiding efficiency values as it likely drives the stimulation deeper towards the sacral (S2-4) micturition center. This stimulating array was then tested systematically with five UB-scES frequencies (5, 15, 30, 45 and 60 Hz) during six fill/void reflex cycles (one cycle without stimulation) and repeated in two separate urodynamic sessions for a total of 18 cycles. The results are illustrated graphically in Fig. 2, with the means from the three separate urodynamic sessions for six fill/void reflex cycles represented. Frequency, varied at sub-motor threshold voltage levels, was most effective for voiding efficiency at 30 Hz (88.1 ± 1.1%). Importantly, across all 18 fill/void cycles and frequencies tested, detrusor leak point pressure (22.1 ± 4.0 cmH_2_O) and maximum detrusor pressure (29.0 ± 4.0 cmH_2_O) were both within recommended guidelines established for upper and lower urinary tract preservation^28^. When scES was not used, bladder pressure also remained within normal limits (24.3 ± 14.2 cmH_2_O). Note that the one cycle from each session that was performed without the use of scES had the lowest efficiency (8.3 ± 4.6%).

**Figure 3:**
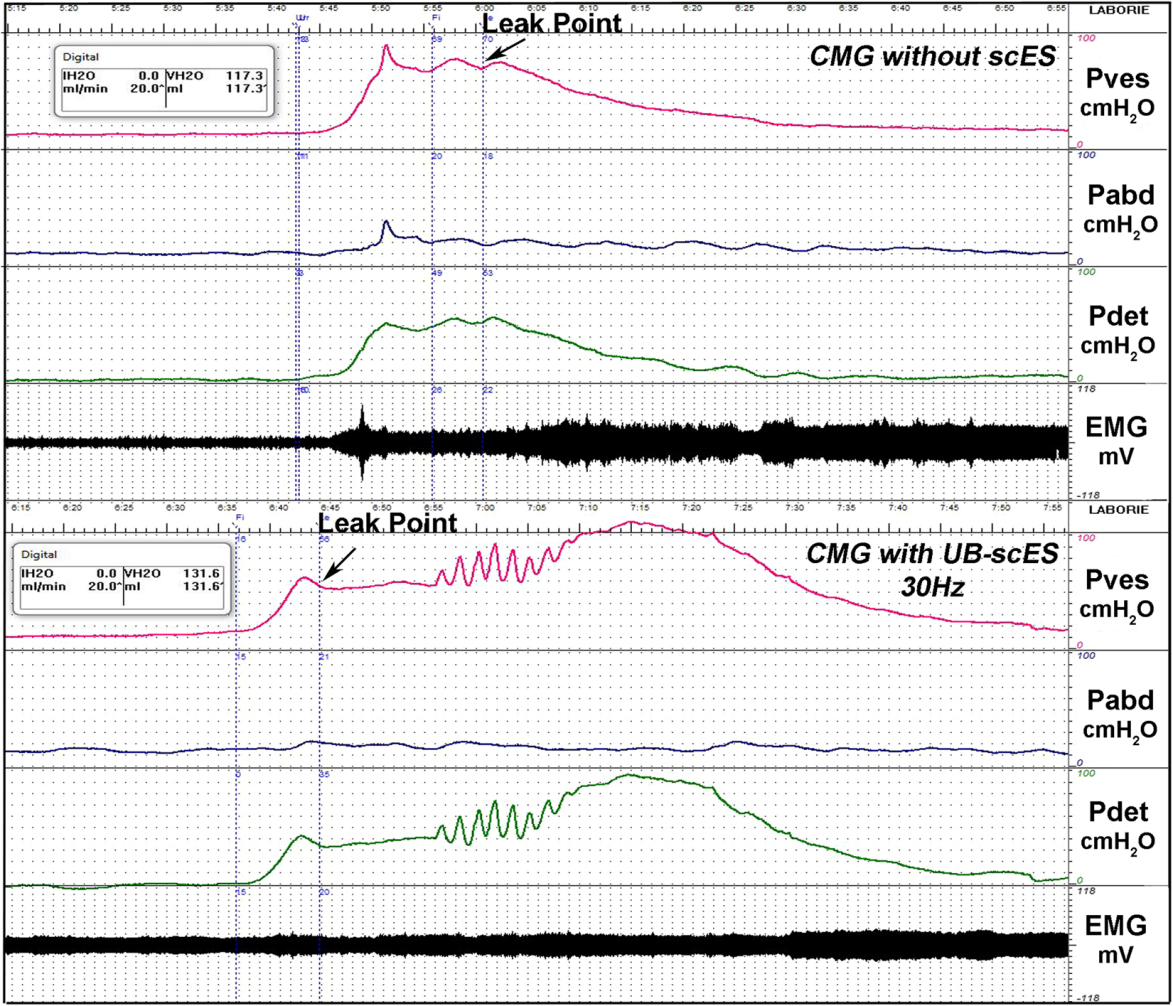
Cystometrogram raw recordings with and without scES. An example of original cystometry recordings without scES and with UB-scES at 30 Hz. UB-scES resulted in an increase in the area under the contraction curve, contraction duration, as well as filling capacity as compared to filling without scES. Differential patterns of EMG activity were noted during filling and compared using the same gain settings across recordings. The bladder contraction during cystometry without scES coincided with an increased pattern of EMG activity as compared to UB-scES at 30 Hz. Note that the fill volume without stimulation was 117.3 ml (capacity of 137 ml and a post-void residual of 132 ml) and 131.6 ml with stimulation (capacity of 139 ml and a post-void residual of 14 ml).

The area under the contraction curve was assessed for differences between filling without scES relative to UB-scES at 30 Hz (2239.3 ± 373.4 vs 2647.8 ± 1692.9 cmH_2_O^2^). Area under the curve, the contraction duration, and filling capacity trended toward an increase with UB-scES at 30 Hz (see example in Fig. 3); however, the average increase in these parameters across all trials at 30 Hz alone as well as across each frequency tested was not significant when compared to cystometry without scES, likely due to an n = 1 in this portion of the study. The range of capacity values were relatively similar across frequencies tested and as compared to no scES: 121.0 ± 7.8 ml − 5 Hz; 139.3 ± 11.0 ml − 15 Hz; 113.3 ± 13.6 ml − 30 Hz; 102.3 ± 24.3 ml − 45 Hz; 125.0 ± 14.4 ml − 60 Hz versus 111.0 ± 13.4 ml − no scES. Area under the curve demonstrated more variability across all other frequencies tested (3282.7 ± 1177.8 − 5 Hz; 2531.3 ± 765.5 − 15 Hz; 2916.0 ± 636.4 − 45 Hz; 2058.8 ± 40.6 − 60 Hz). The greater area evident at 5 Hz stems from a sustained bladder contraction during one fill/void cycle that resulted in a minimal leak (35.8 ± 9.6%). Surface EMG activity obtained during B23’s cystometry using UB-scES displayed a quiescent pattern of activation timed to the detrusor contraction (peak of 23.7 mV) compared to an asynchronous firing pattern (peak of 80.3 mV) that would limit emptying during cystometry without scES (Fig. 3). Note that electrode configurations in both the upper lumbar (5−/0−/6−/11−/1−/12−//7+/2+/13+) and middle lumbar (7−/2−/8−/13−//3+/9+/14+/1+/6+/12+) regions of the array were assessed with repeated cystometry and did not result in greater gains in voiding efficiency as compared to the lower lumbosacral region for this research participant [57.9 ± 21.1% (upper) and 68.5 ± 12.2% (middle) versus 88.1 ± 1.1% (lower)].

**Figure 4:**
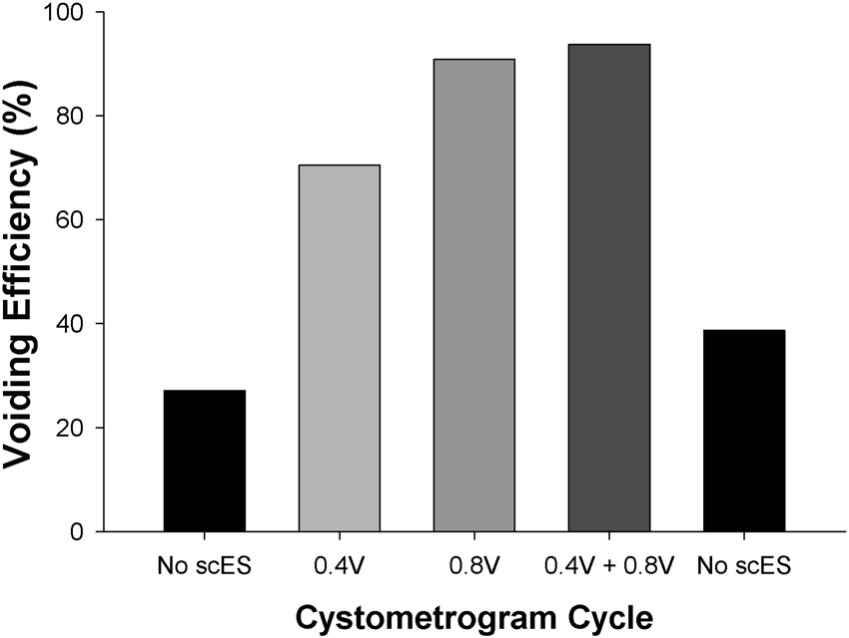
Voiding efficiency with sub-motor threshold voltage variation. Bladder voiding efficiency responses to varying sub-motor threshold voltages shows the greatest improvement at a higher threshold alone or in combination with a ramped stimulation program (see text). The final fill/void cycle demonstrates a return toward the initial, pre-stimulation voiding efficiency value, indicating a positive overall effect of stimulation alone on bladder emptying [Electrode configuration used: L5/S1 region (10−/4−/15−/9−//3+/8+/14+), 30 Hz, 450 µs).

Research Participant B23 returned for an additional evaluation 6 months after completing the bladder mapping study. The same location/electrode numbers and optimal frequency (30 Hz; per Fig. 2) were used during this assessment except voltage was varied (sub-motor threshold 0.8 V and half the intensity, 0.4 V). The sequence for five fill-void cycles during the 90-minute session was no scES, 0.4 V, 0.8 V, an assessment of the dual program RestoreAdvanced capability (Program 1 at 0.4 V during filling then changing to Program 2 at 0.8 V upon onset of urge to mimic the concept of having one ongoing set of parameters for storage and then switching to void parameters at an appropriate time), followed by a second cycle with stimulation off. The first fill/void cycle performed without the use of scES yielded a low voiding efficiency (27.2%) (Fig. 4). The voiding efficiency outcome was 0.8 V > 0.4 V > no scES. Having a lower threshold (0.4 V) running continuously and immediately ramping to a higher threshold once desire to void was recognized did not alter the voiding efficiency to the 0.8 V stimulus. Interestingly, bladder capacity was not altered with this sequence of repetitive fills and scES (103 ml, first cycle versus 98 ml, fifth cycle), indicating the effect was limited to bladder emptying. The last fill/void cycle without scES demonstrates the effect of stimulation alone as voiding efficiency returned toward the initial pre-stimulation value.

**Figure 5:**
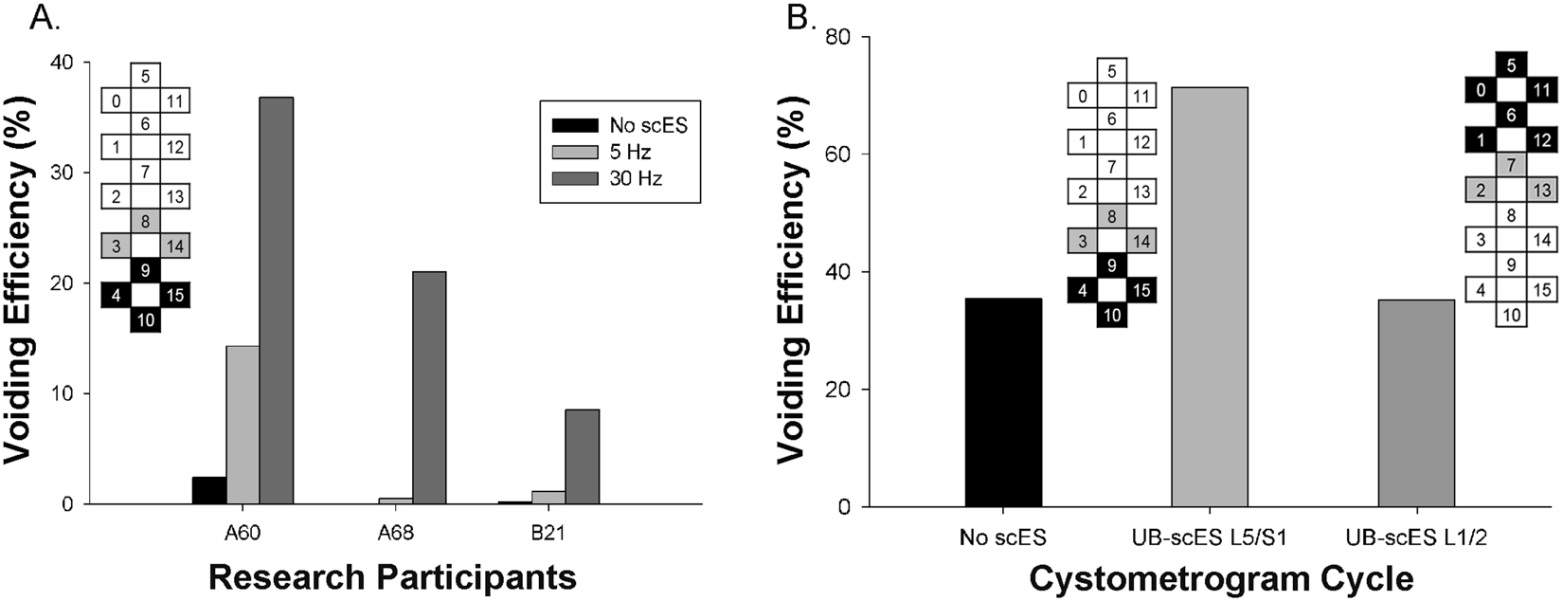
UB-scES conducted in four additional participants improves voiding efficiency using the effective stimulation configuration. (**A**) The stimulation configuration found effective for voiding efficiency (left inset, 10−/4−/15−/9−//3+/8+/14+) in B23 was replicated in both motor/sensory complete and sensory incomplete SCI participants, revealing 30 Hz was sufficient to induce improved bladder emptying as compared to a lower frequency or no UB-scES. The greatest improvement in voiding efficiency was apparent in the individual (A60) who demonstrated partial reflexive bladder emptying without stimulation versus those individuals having little to no bladder emptying without stimulation. (**B**) The lower (L5/S1, 10−/4−/15−/9−//3+/8+/14+) and upper (L1/L2, 5−/0−/6−/11−/1−/12−//7+/2+/13+) regions of the lumbosacral cord were targeted in a fourth participant (A41 - C4, AIS A) during his follow-up urodynamic assessment (post-stand training) with UB-scES, demonstrating the lower lumbosacral region was effective for bladder emptying (as found with mapping in B23). On the electrode array diagrams, cathode selection (−) is indicated in black, anode selection (+) in grey, and the inactive electrodes in white.

To examine the efficacy of the lower lumbosacral configuration found to be effective for voiding in B23, UB-scES using that configuration was conducted in four additional research participants during their urodynamic assessments (note that the three Fig. 5A participants all perform intermittent catheterization for bladder management, whereas the Fig. 5B participant has a suprapubic catheter). These four participants were male, with an average 6.5 ± 1.9 years post-injury (at the time of the post-training urodynamic assessment). They each were participating in the scES studies assessing the impact of task-specific training (step-scES, stand-scES, voluntary motor training – Vol-scES, and cardiovascular training – CV-scES) on both the motor and autonomic systems. The first fill/void cycle for each cystometry event was performed without the use of scES, with little to no leak in each participant (Fig. 5A). For three of the four participants, three fill/void cycles were examined in a 90-minute session. The effective electrode configuration for research participant B23 was tested during the second and third fill/void cycle using two different frequencies, 5 Hz and 30 Hz. Note that an increase in voiding efficiency was demonstrated again with scES, with 30 Hz > 5 Hz, which occurred in both AIS A and B participants. In a fourth participant (Fig. 5B, A41, AIS A and a neurological level of injury at C4), the lower end of the electrode array (L5/S1) was targeted during urodynamics using the effective voiding efficiency configuration as well as the upper end of the electrode array (L1-L2). The first fill/void cycle was performed without the use of scES, demonstrating low voiding efficiency output. The next fill/void cycle utilized the same effective lower lumbosacral configuration and stimulation parameters (30 Hz, 450 µsec) as B23, resulting in a doubling of the initial, pre-stimulation voiding efficiency value. The last fill/void cycle targeted the upper lumbar region using multi-electrode configurations (30 Hz, 450 µsec) focusing on the L1/2 region, resulting in low voiding efficiency, equivalent to the pre-stimulation voiding efficiency value.

## Discussion

Given the initial participant’s relatively small bladder capacity due to the presence of a suprapubic catheter as well as initial bladder emptying gains with LT alone and in combination with UB-scES, the focus of the current mapping study was on improving voiding efficiency. Multiple consistent and repeated fill/voiding cycles could be performed during Urodynamics due to the participant’s low capacity, facilitating the investigation of potential configurations and effective stimulation parameters. The objective was to determine if an effective electrode configuration and stimulation parameter(s) could be achieved with UB-scES to promote more efficient bladder emptying in persons having a motor complete SCI. Mapping for bladder function with different electrode configurations during repeated cystometry revealed increases in the efficiency of the reflexive void with values within recommended clinical guidelines. The electrode combination at the lower end of the stimulator array (L5/S1 region – (10−/4−/15−/9−//3+/8+/14+), optimized at 30 Hz in one individual, was then tested in four more individuals who showed improvements in bladder emptying as well. However, three of the four participants never demonstrated voiding efficiency greater than 50%, one as low as 10% indicating that the results are highly patient specific (Fig. 5A).

Initial use of scES at the Kentucky Spinal Cord Injury Research Center, University of Louisville targeted improvements in stepping, standing, and voluntary movement in response to provided task-specific sensory cues in motor complete SCI^16,20^. Unexpected off-target gains to other physiological systems such as bladder, sexual function, and temperature regulation were also evident^16^. It is important to note that these multiple autonomic changes developed even though the stimulation parameters were aimed at influencing the motor system and the execution of specific motor tasks. Based on these outcomes, we proceeded to systematically and objectively evaluate participants via urodynamic assessments prior to and following task-specific training interventions, including the use of scES. We have previously shown that locomotor training alone was sufficient to induce significant improvements in multiple bladder parameters, such as increased capacity, voiding efficiency, detrusor contraction duration as well as decreased detrusor leak point pressure^15^. These overall urological improvements reported previously also include participant B23. We now demonstrate in this study that the use of scES alone, without additional training, can promote increases in voiding efficiency. An effective stimulation frequency and electrode configuration at the lower end of the stimulator array over the L5/S1 region yielded voiding efficiency values close to the standard threshold of 90%^28^, or even better (as seen for B23 in Fig. 4, at 93.8%). Although voiding efficiency shifted closer to normal in the four participants who were later assessed, the configuration used in these assessments was one deemed effective for B23. Note, three of the five participants exhibited much more reduced voiding efficiency responses than B23. We know from mapping studies for stand and step training interventions that optimal configurations vary from individual to individual, necessitating mapping^20^.

Over the years, various modes of electrical stimulation have been used to treat different lower urinary tract dysfunctions. In comparison to scES used in this study, the Finetech-Brindley device (marketed in the U.S. as VOCARE™ Bladder System) is also an implanted electrical stimulation device, targeting the anterior sacral roots (S2–S4), and has shown positive results for bladder control and quality of life in individuals having SCI^29–31^. The device has been used over the years to aid bladder emptying via intermittent stimulation bursts that maintain a constant bladder pressure while periodically relaxing the urethral sphincter, allowing urine to flow in between stimulus intervals (post-stimulus voiding)^32,33^. The implantation procedure is usually combined with a posterior sacral rhizotomy to abolish hyperreflexive bladder and sphincter contractions as well as autonomic dysreflexia, triggered by bladder fullness. Despite the clinical improvements in bladder function and beneficial off-target effects in some individuals on enhancing bowel emptying and evoking penile erections, one of the major accompanying limitations of the Finetech-Brindley device as well as a decline in use over the years stems from the irreversibility of ablating intact neural roots and thus, the potential for future treatment options^34^.

Peripheral nerves have also served as promising targets to influence the bladder circuitry after SCI, where stimulation to a particular nerve or nerve branch may provide more specificity and improve mechanistic understanding of differential stimulation approaches. For instance, pudendal afferents activated with surface electrodes have been shown to suppress detrusor hyperreflexia and instability, and include such methods as dorsal penile nerve stimulation^35–37^, intraurethral stimulation^38^, as well as anal and vaginal plug electrodes^39–41^. Stimulation of pudendal afferents and branches thereof have also been successful in improving both the storage and emptying phases of micturition in animal models^42,43^ and in humans^44,45^. As an important translational application in SCI, pudendal nerve stimulation does not appear to be dependent on descending input, as reflex bladder inhibition (continence response) achieved with low frequency stimulation was preserved after spinal transection^42,43^. In a similar manner to what we report on in the current study, pudendal nerve stimulation at higher frequencies (20–50 Hz) has been found to be effective at promoting bladder contractions without co-activation of the external urethral sphincter, thus enhancing the emptying phase of micturition^42,45–47^.

From a clinical and research perspective, other methods of influencing the bladder circuitry to provide greater control over urinary tract function have also focused on reducing bladder overactivity. In terms of improving the storage phase and symptoms related to urinary frequency and urge incontinence, use of continuous sacral neuromodulation (applied most commonly over S3 nerve root) has been shown to be effective for improving continence, traditionally in individuals with non-neurogenic bladders having overactive bladder syndrome and who did not respond to conservative therapies, such as biofeedback, behavioral therapy, and pharmacological options^48,49^. Additional studies with sacral nerve stimulation in the SCI population have shown that improvements in bladder capacity and incontinence were most effective in incomplete individuals as compared to those with complete SCI^50–52^, suggesting the influential role of preserved spinobulbospinal pathways contributing to modality effectiveness^53^.

Similar to sacral neuromodulation, percutaneous tibial nerve stimulation (accessed just over the medial malleolus) has been reported to relieve symptoms related to bladder overactivity, improving primarily the storage phase of micturition^54,55^. In some studies, application of tibial nerve stimulation has been used effectively in suppressing neurogenic detrusor overactivity in multiple sclerosis patients^56,57^, suggesting its potential utility in SCI. However, in another study of MS patients with over active bladder syndrome, no evidence of an acute effect on inhibiting bladder activity was seen^58^. Furthermore, a recent study in cats demonstrated that the effect of bladder inhibition with tibial nerve stimulation was abolished following a spinal transection, suggesting a dependent role upon supraspinal input for eliciting a positive effect and thus, perhaps not suitable for complete SCI^59^.

Even though percutaneous tibial nerve stimulation is less invasive than sacral neuromodulation, the stimulation site is at a distance from the central nervous system and involves repeated application and thus the long-term durability may not be sustained^60^.

The use of scES described in the current study may be an effective alternative approach which mechanistically may involve indirect activation of the same neural networks for bladder function. However, it remains to be shown if some features like incontinence management will occur with scES. Also, the current results of low voiding efficiency in three out of the five participants will require further investigations. Although scES requires implantation surgery, which may be a drawback for some individuals to consider, given that the consequences of SCI affect multiple systems, this intervention may also benefit other autonomic systems controlling cardiovascular, respiratory, bowel, sexual function and temperature regulation. Thus, the potential multi-system benefits of scES have the capability for dramatically impacting quality of life. Furthermore, once a participant’s device is programmed with effective stimulation programs, the ability for on-demand device use becomes particularly essential for initiating particular tasks, such as triggering the voiding phase of micturition.

Although the mechanisms associated with the improvements in voiding efficiency shown here are not entirely known, optimizing the level of excitability of the nervous system through scES may foster a priming effect at the spinal cord, thus modulating the excitability of spinal reflexes. The ability of the spinal cord to interpret both incoming sensory input and residual descending drive with sufficient responses to that information is important in this regard. The central activation and excitation driven by the scES parameters may influence neural output to the detrusor muscle, causing a more sustained contraction in comparison to the quick bursting contractions that are typical of hyperreflexia which limits bladder emptying. Modulation of reflex mechanisms controlling micturition can arise from spinal convergence of somatosensory input leading to a suppression of the bladder guarding reflex and resulting in decreased urethral sphincter contractions and improved voiding efficiency^61^. The bladder is also a unique visceral organ in that, in addition to various reflex mechanisms that exist to modulate both the storage and voiding phases^62^, it also exhibits predominately voluntary regulation, unlike other visceral organs such as the heart and gastrointestinal tract, which receive tonic neural control. With ample descending drive, scES may provide a means of coordinating both storage and voiding reflexes (including sphincter tone) while ultimately promoting a decreased pressure system for the lower urinary tract.

A potential limitation with scES is that the stimulation configuration we found effective for voiding efficiency may not be as effective for the other phase of micturition (storage). Participant B23’s bladder capacity was evaluated at each urodynamic assessment, with and without stimulation. While we did see slight increases in capacity in the presence of UB-scES, the improvement was not consistent. This may be expected, as the participant’s bladder capacity was already limited due to continual drainage and lack of expansion from the presence of a suprapubic catheter. In addition to targeting the upper electrode array (sympathetic output to bladder), the effect on bladder capacity may also be frequency-dependent. Studies in a feline model of chronic SCI demonstrate improvements in bladder capacity utilizing low frequency stimulation through an inhibitory pudendal-bladder reflex^42,43,63^. It is important to also note that repeated cystometry, as conducted here during mapping, can result in a sequential increase in bladder volumes, thus influencing post-void residual values. Even though we report no significant improvements in bladder capacity across the representative testing trials for B23, frequency was also varied in reverse order (i.e. starting at 60, 45, 30, 15, 5 Hz, and no scES), as well as testing in a random order, also resulting in no appreciative changes in bladder capacity in this individual (data not shown). Importantly, varying the order of test trials also did not lead to subsequent increases in voiding efficiency.

If voiding with UB-scES can be achieved, residual volumes may not be low enough to avoid catheterization, although the number of times could still be reduced, perhaps just to the morning and night-time, giving more flexibility during daily activities and eliminating disruption of sleep. However, regardless of the extent of the effect that will be obtained, any improvement in bladder function, even incremental, would have a dramatic impact on health and quality of life for those suffering the lifelong consequences of neurologic injury. Moving the electrode array one or two segments lower to infringe directly upon the spinal micturition center (S2-4) is a viable alternative for future projects. However, the overall concept of the epidural projects for our center with the currently available technology is to use multiple configurations for multiple systems, so moving the placement even one segment could impact the positive benefits seen to date on the cardiovascular and respiratory system^64,65^.

## Conclusions

SCI disrupts normal control of bladder function by interrupting both afferent transmission to higher centers and efferent drive to lower spinal levels that modulate output to the lower urinary tract. As a result, aberrant reflexes develop below the level of a spinal lesion to produce uncoordinated activity leading to incontinence, inefficient bladder emptying and high pressure. This study provides proof of principle evidence to show that scES targeting the parasympathetic outflow to the bladder was sufficient to induce consistent increases in voiding efficiency, perhaps influencing detrusor contraction strength and external urethral sphincter relaxation. Replication of this effect seen in four additional participants warrants further investigation. Future work could involve determining the efficacy by applying two types of stimulation, perhaps each running at different frequencies, and whether if used concurrently would affect both stages of micturition. Extending the length of the electrode array may also provide more opportunity for effective stimulation parameters. Electrode location remains an important issue for future optimization. In addition, it will be important to identify the long-term effects of stimulation, whether the effect is sustained or dampens overtime as well as its impact on overlapping systems such as bowel and sexual function. Further, understanding the relationship between lower limb and/or abdominal muscle activation and detrusor and/or external urethral sphincter contraction can aid in the identification of optimal configurations.
